# Augmented Reality to Assist Skin Paddle Harvesting in Osteomyocutaneous Fibular Flap Reconstructive Surgery: A Pilot Evaluation on a 3D-Printed Leg Phantom

**DOI:** 10.3389/fonc.2021.804748

**Published:** 2022-01-06

**Authors:** Laura Cercenelli, Federico Babini, Giovanni Badiali, Salvatore Battaglia, Achille Tarsitano, Claudio Marchetti, Emanuela Marcelli

**Affiliations:** ^1^ eDIMES Lab - Laboratory of Bioengineering, Department of Experimental, Diagnostic and Specialty Medicine, University of Bologna, Bologna, Italy; ^2^ Maxillofacial Surgery Unit, Head and Neck Department, IRCCS Azienda Ospedaliera Universitaria di Bologna, Department of Biomedical and Neuromotor Sciences, Alma Mater Studiorum University of Bologna, Bologna, Italy; ^3^ Maxillofacial Surgery Unit, Policlinico San Marco University Hospital, University of Catania, Catania, Italy

**Keywords:** augmented reality, virtual planning, 3D printing, Head and Neck Cancer, Microsoft HoloLens, registration, reconstructive surgery, 3D modeling

## Abstract

**Background:**

Augmented Reality (AR) represents an evolution of navigation-assisted surgery, providing surgeons with a virtual aid contextually merged with the real surgical field. We recently reported a case series of AR-assisted fibular flap harvesting for mandibular reconstruction. However, the registration accuracy between the real and the virtual content needs to be systematically evaluated before widely promoting this tool in clinical practice. In this paper, after description of the AR based protocol implemented for both tablet and HoloLens 2 smart glasses, we evaluated in a first test session the achievable registration accuracy with the two display solutions, and in a second test session the success rate in executing the AR-guided skin paddle incision task on a 3D printed leg phantom.

**Methods:**

From a real computed tomography dataset, 3D virtual models of a human leg, including fibula, arteries and skin with planned paddle profile for harvesting, were obtained. All virtual models were imported into Unity software to develop a marker-less AR application suitable to be used both *via* tablet and *via* HoloLens 2 headset. The registration accuracy for both solutions was verified on a 3D printed leg phantom obtained from the virtual models, by repeatedly applying the tracking function and computing pose deviations between the AR-projected virtual skin paddle profile and the real one transferred to the phantom *via* a CAD/CAM cutting guide. The success rate in completing the AR-guided task of skin paddle harvesting was evaluated using CAD/CAM templates positioned on the phantom model surface.

**Results:**

On average, the marker-less AR protocol showed comparable registration errors (ranging within 1-5 mm) for tablet-based and HoloLens-based solution. Registration accuracy seems to be quite sensitive to ambient light conditions. We found a good success rate in completing the AR-guided task within an error margin of 4 mm (97% and 100% for tablet and HoloLens, respectively). All subjects reported greater usability and ergonomics for HoloLens 2 solution.

**Conclusions:**

Results revealed that the proposed marker-less AR based protocol may guarantee a registration error within 1-5 mm for assisting skin paddle harvesting in the clinical setting. Optimal lightening conditions and further improvement of marker-less tracking technologies have the potential to increase the efficiency and precision of this AR-assisted reconstructive surgery.

## 1 Introduction

Augmented reality (AR) in medicine is a technology that expands on image-guided surgery, allowing intraoperative guidance and navigation. This technique integrates imaging information with the real-world surgical field to give the surgeon a sort of “x-ray vision”.

In recent years, AR technology has been proposed and applied in neurosurgery (1, 2), urology (3–5), orthopedics (6, 7) and craniomaxillofacial surgery (8, 9), among others.

A variety of technologies including traditional projectors, mobile devices such as tablets and smartphones, and head mounted displays (HMDs) have been proposed and used to perceive the augmented surgical field (8, 10). Specifically, several commercial optical see-through HMDs, such as Microsoft HoloLens (Microsoft, Redmond, WA) and Google Glass (Google Inc., Mountain View, California, USA), have gained broad availability and are being explored for applications in surgery. However, a small number of AR-based solutions for intraoperative surgical guidance have been successfully demonstrated in humans, e.g. for spine and hip surgery (11–13), while other promising solutions have been described and demonstrated on phantom (14–16).

In craniomaxillofacial surgery, the AR technology can be considered an evolution of the navigation-assisted surgery, and it represents a promising tool in aiding complex surgical procedures, such as mandible reconstruction with fibula flap, with potential to avoid or limit the use of cutting guide technology. The osteomyocutaneous microvascular fibular flap harvesting represents the reconstructive gold standard for complex mandibular defects resulting from tumor resections, trauma or malformations (17). For these cases, the use of 3D technologies and computer-aided design/computer-aided manufacturing (CAD/CAM) is essential to provide an accurate planning and to enhance the quality of the surgical outcomes. While CAD/CAM has become a quite common practice to virtually plan the bony resection and reconstruction, for skin paddle incision the surgeon still relies on measurements made on radiological imaging which are then reported on the patient’s skin. Besides computerized tomographic angiography and magnetic resonance tomography, Doppler sonography is an affordable and harmless method commonly used to preoperatively determine arterial supply to the lower extremity. i.e. to identify the cutaneous perforators in fibula osteocutaneous free tissue transfer patients (18–20).

Only few recent experiences reported the use of accurate virtual planning and CAD/CAM technology for skin paddle harvesting and localization of the cutaneous perforator vessels of the fibula vascular anatomy for supplying the free flap (21, 22). In this field, the AR technology may offer an alternative or combined approach to assist intraoperatively the surgeon in skin paddle harvesting.

It would be possible to plan and reproduce the soft tissue resection and reconstruction and not only the bony part of the 3D-aided surgery. In order to achieve this goal, once the planning is carried out either on the resection and on the fibular skin area planned for soft tissue reconstruction, the reconstructed 3D virtual plan may be transferred to AR technology. This will give the surgeon the opportunity to reproduce the planned reconstruction, based on the three-dimensional position and reciprocal position to the bony segments and flap insetting, in restoring the defect.

As great advantage, the AR technology, when deployed on a wearable head mounted display, allows the direct view of the planned resection and reconstruction on the surgical field without the need for the surgeon to alternate viewing between the surgical field and external monitors such as in the case of standard navigation systems, with potential clinical benefits of reducing operative time and improving surgical outcomes. We have recently reported preliminary case series where the feasibility of a proof-of-concept AR based protocol implemented on a tablet for assisting procedures of free fibula bone harvest (23) and of galeo-pericranial flap harvest (24) was demonstrated. In those experiences the AR guidance was based on a marker-less registration, i.e. without the need of invasive placement of fixed fiducial markers on the patients. However, the provided registration accuracy between the real and the virtual content, that will affect the reliability of the virtual planning overlaying the patient anatomy, requires to be systematically evaluated before widely promoting this AR protocol in the clinical practice. Indeed, virtual-to-real scene registration, patient position tracking and projection of the digital content onto the targeted anatomical structures are crucial steps of AR-guided navigation systems (8, 25, 26).

In this paper, we describe the AR based protocol we developed for assisting skin paddle harvesting in osteomyocutaneous fibular flap reconstructive procedure, usable both with a handheld device, such as a tablet, and with a HMD, such as Microsoft HoloLens 2 smart glasses. The study was also designed to evaluate the registration errors associated with the two display solutions, and the achievable success rate when simulating the AR-guided task of skin paddle harvesting on a 3D printed human leg phantom.

## 2 Materials and Methods

The study was designed in order to first implement the AR based protocol on both the tablet and the HoloLens 2 smart glasses. Then, a first testing session to quantify the achievable registration accuracy with the two display solutions, and a second testing session to evaluate the success rate in executing the AR-guided skin paddle incision task on a 3D printed leg phantom were performed.

In the following sections the development and implementation phase, as well as the experimental phase of the study were reported.

### 2.1 Development Phase

**Figure 1:**
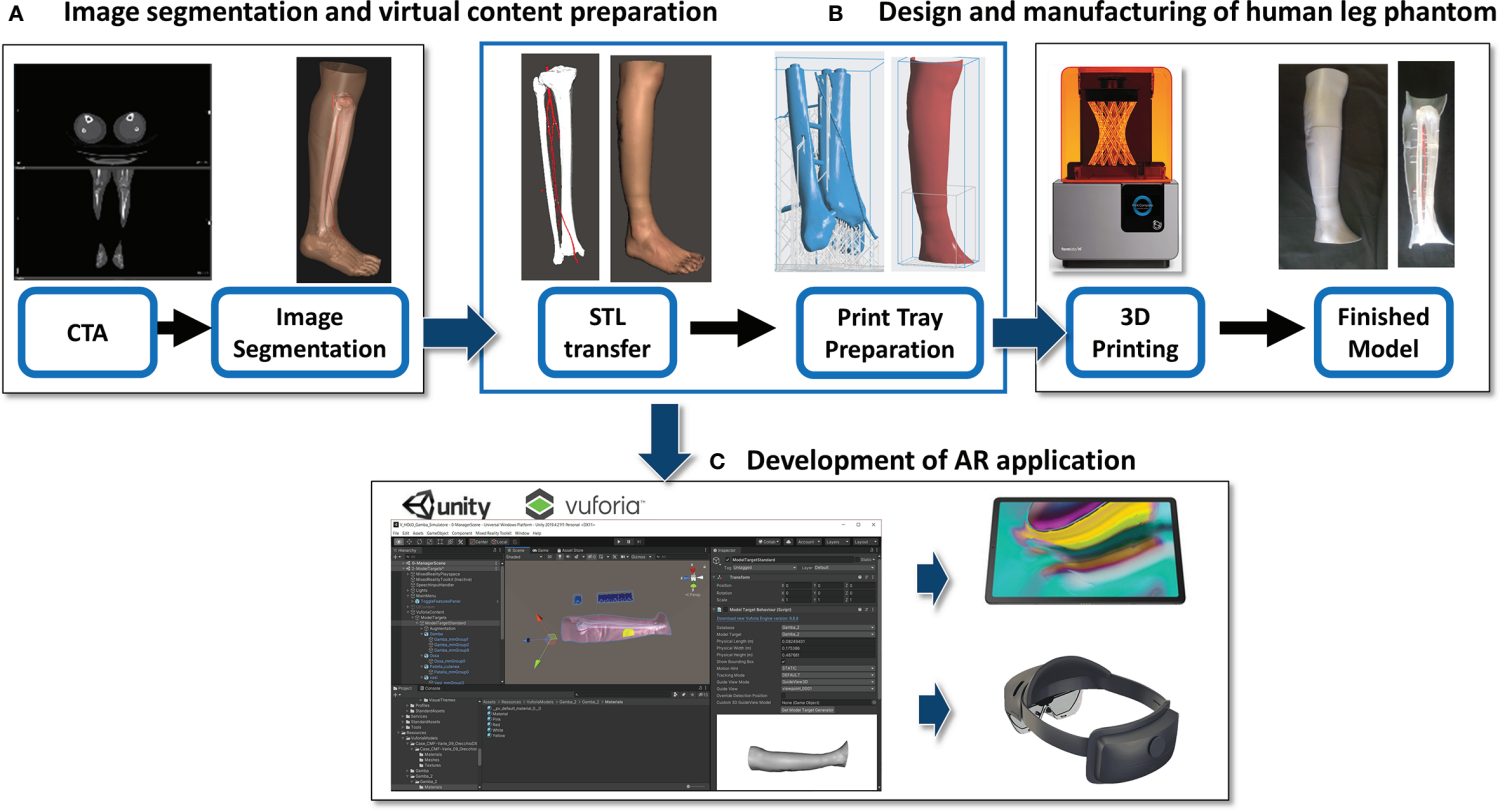
Description of the development work flow. **(A)** Image segmentation and virtual content preparation; **(B)** Design and manufacturing of the human leg phantom; **(C)** Development of the AR application.

This phase consists of three steps: A) Image segmentation and virtual content preparation; B) Design and manufacturing of the human leg phantom; C) Development of the AR application (**Figure 1**).

#### 2.1.1 Image Segmentation and Virtual Content Preparation

The process started from acquisition of real computed tomography angiography (CTA) datasets of a patient lower leg, which represents the donor-site for osteomyocutaneous fibular flap harvesting procedure. CTA scans were acquired after administering nonionic contrast media intravenously (Xenetix 350 Guerbet) and with a slice thickness of 0.6 mm (Lightspeed VCT LS Advantage 64 slices; General Electric Medical System).

Anatomical areas of interest of the subject’s leg were segmented using D2P™ software (3D Systems Inc., Rock Hill, SC, USA): bones (tibia and fibula), arterial vessels (popliteal, fibular, tibial and perforating arteries) and leg skin (distinguishing the skin paddle profile for harvesting, according to the virtual planning for mandibular reconstruction).

Three-dimensional meshes were then generated from all the segmented masks, and saved in STL format.

#### 2.1.2 Design and Manufacturing of Human Leg Phantom

From the virtual models obtained in the previous step, a tangible phantom made of photosensitive resin was produced *via* a stereolithography (SLA) 3D printer (Form 3, Formlabs, Somerville, MA, USA).

**Figure 2:**
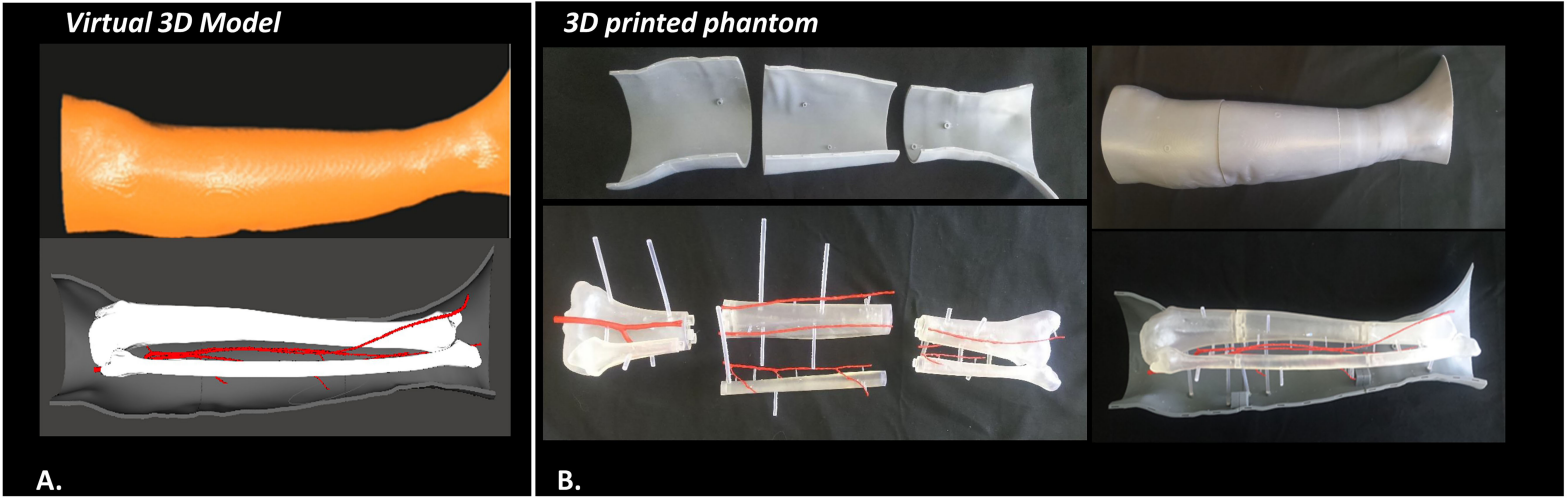
Virtual **(A)** and 3D printed **(B)** model of the designed human leg phantom, used for the experimental phase.

To make the virtual leg model compatible with the build volume of Form 3 printer (14.5 × 14.5 × 17.5 cm), CAD processing was carried out using MeshMixer software (Autodesk Inc., CA, US) in order to create a modular phantom composed of several parts. In detail, for the skin layer and the bones/vessels block, three cross sections were designed, each divided into two symmetrical portions defined by a longitudinal cutting plane, and a set of joints among the various separate parts was created. Each component was printed individually, using a grey resin for the skin (4 mm thick shell) and a clear resin for bone and vessel structures. Then, arteries were colored red to differentiate them from bones, and all pieces were assembled (**Figure 2**).

#### 2.1.3 Development of AR Application

The obtained virtual models of the lower leg, i.e. bone anatomy, arteries and the planned skin paddle profile with the corresponding selected perforator vessel, were imported into Unity 3D software (Unity Technologies, San Francisco, CA, USA) extended with a specific software development kit for creating augmented reality apps (Vuforia Engine package, PTC, Inc., Boston, MA, USA).

**Figure 3:**
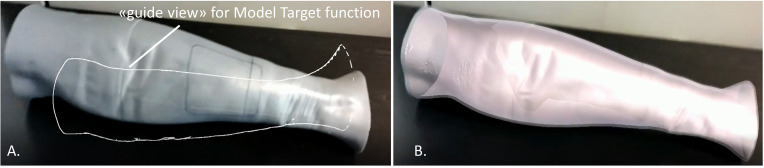
Example of the “guide view” in the Model Target function **(A)**, showing an approximation of the object shape used for tracking the phantom leg and matching to it the virtual content **(B)**.

The tracking algorithm and registration between the virtual content and the real scene were implemented using the “Model Target” function of Vuforia Engine, which allows the marker-less tracking of a physical object in the real world by recognition of the shape of the 3D object itself observed from a certain perspective. Model Target function enables to recognize and track objects in the real world based on their shape. To make a Model Target for a particular object the 3D model data for the object, such as a 3D CAD model or a 3D scan of the object, is necessary. A Model Target requires that the user holds the AR display device at a particular angle relative to the object, and at a particular distance to initialize the tracking. To aid with this process, the application typically draws an image (“guide view”) showing an approximation of the object shape from this distance and viewing angle, so that the user just needs to move the AR display until the object matches this guide view. After that, tracking can begin (**Figure 3**).

**Figure 4:**
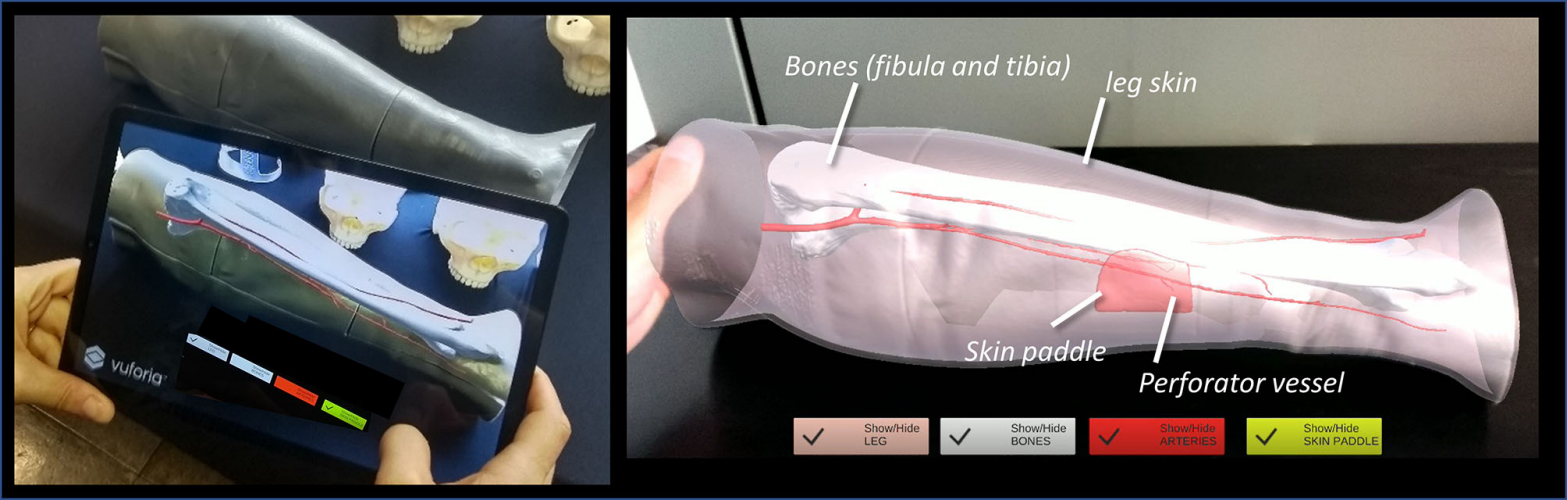
The AR application generating the holographic overlays superimposed on the real phantom anatomy, as displayed to surgeon *via* tablet.

**Figure 5:**
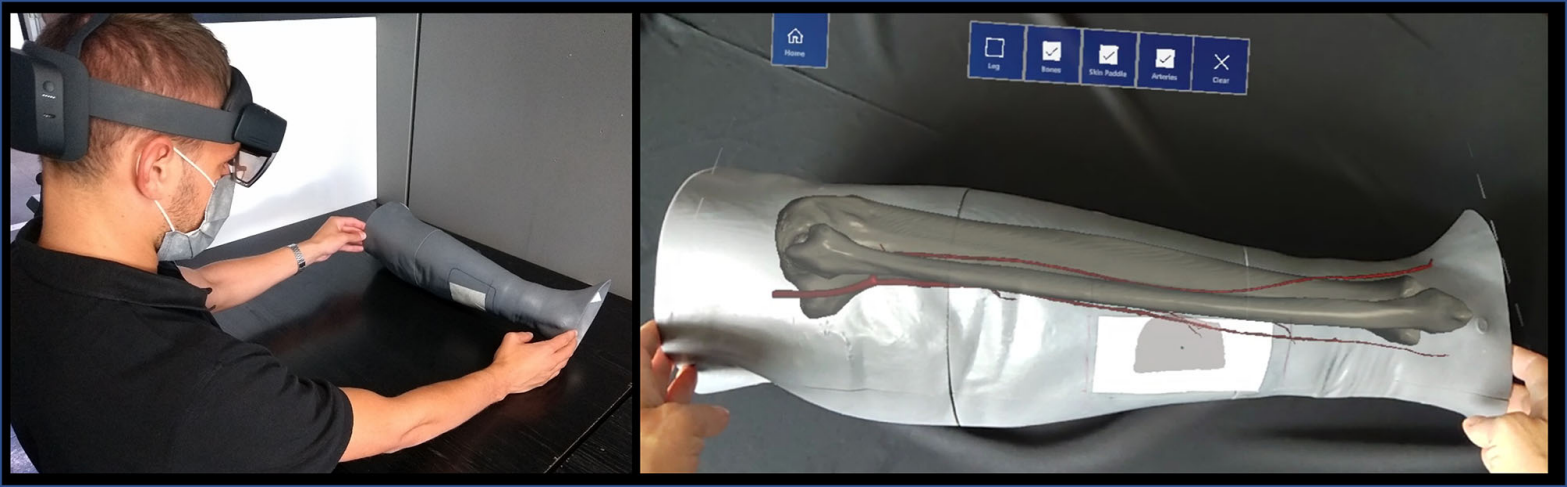
The AR application generating the holographic overlays superimposed on the real phantom anatomy, as displayed to surgeon *via* HoloLens 2 smart glasses.

In this study, the CAD model of the leg skin was used as Model Target for virtual-to-real scene registration. The created AR application was built both as an Android app for mobile devices then deployed on a Samsung Galaxy TAB S5E (**Figure 4**), and as a UWP (Universal Windows Platform) app deployed on Microsoft HoloLens 2 smart glasses (**Figure 5**).

In both cases, the AR application generates “holographic” overlays, by rendering the bony and vascular anatomy of the simulated patient leg, and also the planned skin paddle profile for harvesting to be used as guiding information during the experimental task performed on leg phantom.

Interactable user interface toggles (check boxes) were added to turn off and on the rendering of each virtual anatomical structure (skin paddle, bones, vessels) (see **Suppl_Video_1_Tablet_DEMO** and **Suppl_Video_2Holo_DEMO**).

For the HoloLens application, voice commands to show/hide the virtual anatomical structures were also implemented in order to provide a completely hand-free AR guidance system.

A portable high-performance workstation (Intel(R) Core i7-10750H, CPU@ 2.60GHz, 16GB RAM, NVIDIA GeForce RTX 2070) was used for the virtual content preparation and for development of the AR application that was then deployed and run directly in the tablet or HoloLens 2 smart glasses.

### 2.2 Experimental Phase

Experimental tests on phantom were carried out in the following two phases.

#### 2.2.1 Test Session 1

We evaluated the registration error of the two AR display types (tablet-based and HoloLens-based). For each solution, we tested two lighting conditions: 1) environment illuminated by natural daylight (OFF_lamp); 2) artificial lamp light that points directly on the phantom (ON_lamp).

**Figure 6:**
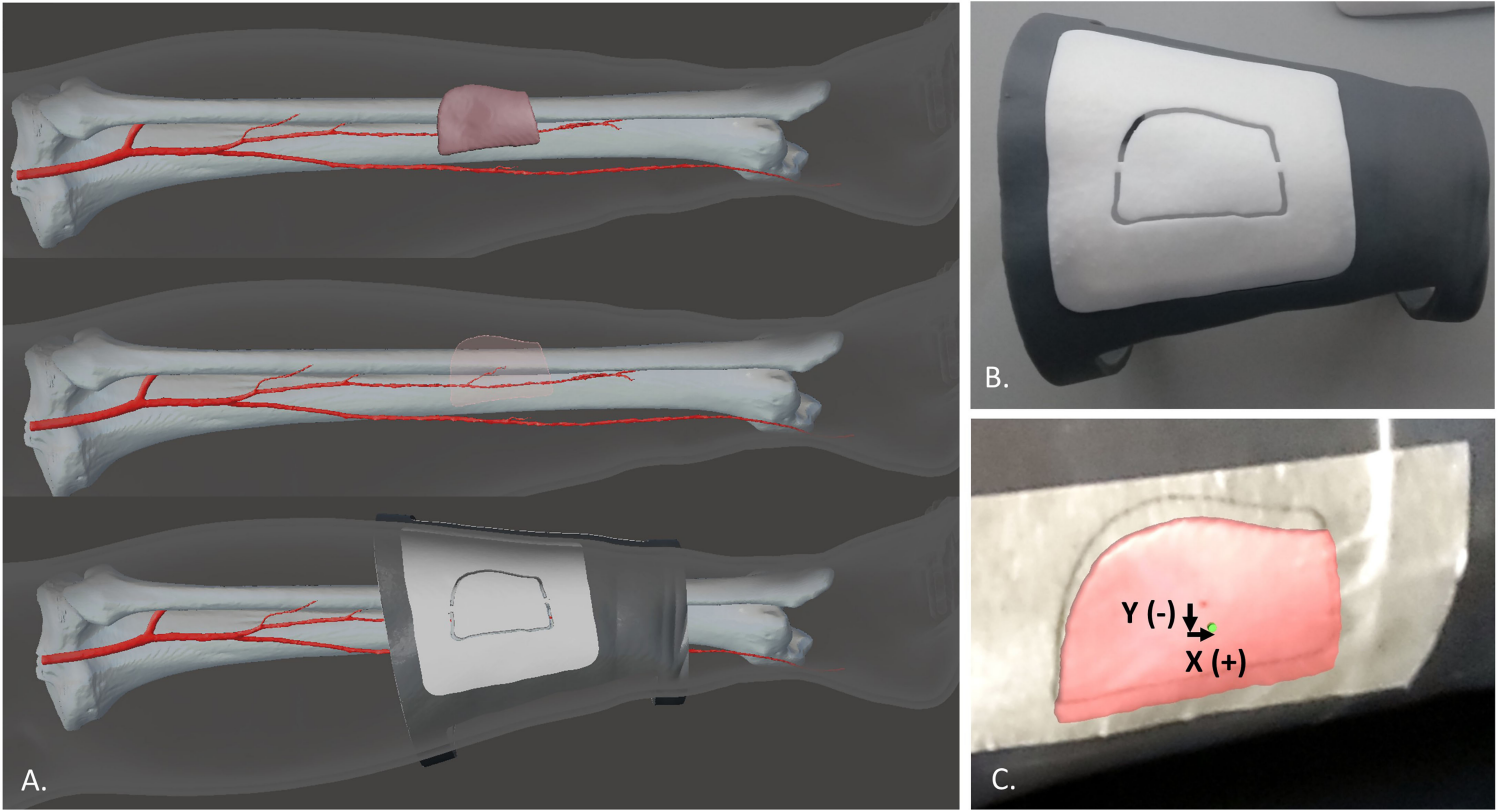
The virtual planning of the skin paddle outline centering the perforator **(A)** and the CAD/CAM template used to transfer this planned skin paddle profile to the 3D printed phantom **(B)**. Comparison between the virtual paddle projected in AR and the real one obtained from the CAD/CAM template applied to the leg phantom **(C)**.

In order to evaluate the registration error, the “real” skin paddle profile and the virtual one projected in AR were compared. The “real” skin paddle profile was obtained from the planned skin paddle: from patient CT angiography the 3D model of the chosen perforator vessel was also reconstructed, then the skin paddle outline was drawn in order to centre this perforator, as we have already described in a previous work (23). Then, we designed and printed a customized guide (template) based on calf proximal and distal diameters, that includes the planned skin paddle outline centering the perforator, and we used this CAD/CAM template to transfer the planned skin paddle profile to the 3D printed phantom (**Figure 6**).

The real profile was obtained by tracing a line on an adhesive tape applied over the leg phantom while following the groove of the CAD/CAM template (**Figure 6B**). The 3D printed template was also provided with a central hole in the paddle (diameter = 1.5 mm) which was used to trace on the tape the central point of the paddle. The same central point was included in the virtual paddle as a green dot. Registration error was quantified as mean deviations between the central point traced on the tape and the virtual one, both in horizontal (X) and vertical (Y) directions (**Figure 6C**). The X, Y deviations were automatically calculated using a Matlab code applied to screenshots acquired for each test. Each testing condition was repeated 12 times and means values ± SD were calculated.

#### 2.2.2 Test Session 2

As second phase, we quantified the success rate in performing on the leg phantom the AR-guided task of skin paddle profile tracing by a group of 8 subjects (5 females and 3 males, aged between 25 and 50, being students, researchers and engineers at University of Bologna, without specific experience with augmented reality systems). Each subject performed the task using both the tablet-based and the HoloLens-based AR application.

**Figure 7:**
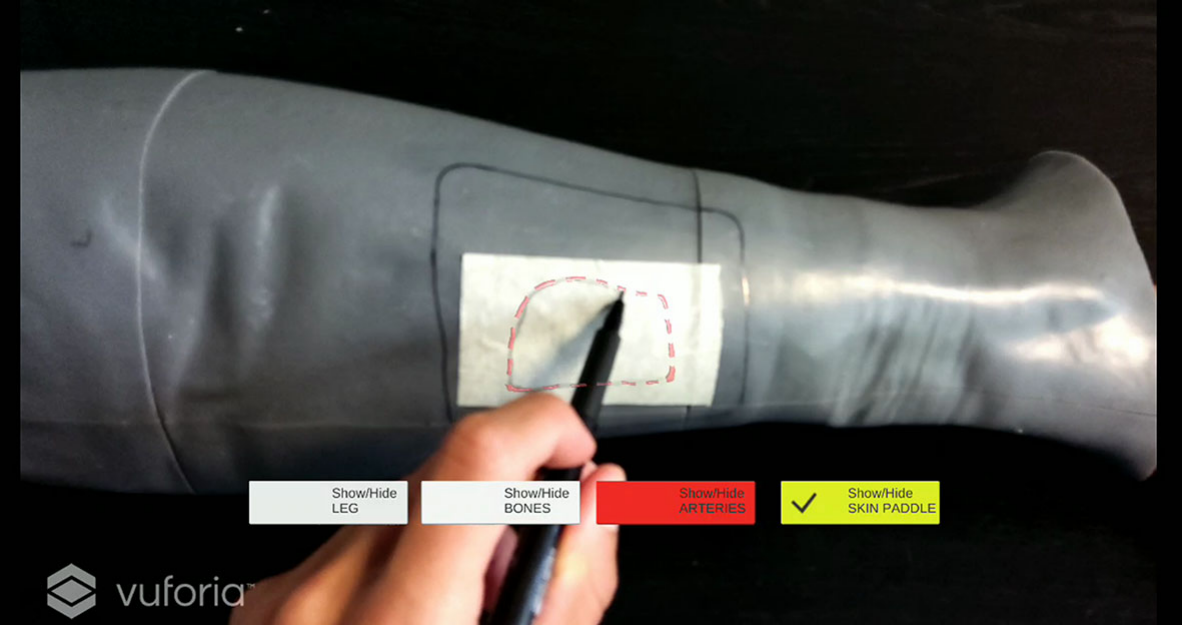
Example of virtual skin paddle profile displayed as a dashed line to avoid occlusion of the real scene by holograms.

The virtual skin paddle profile was designed and displayed as a dashed line to facilitate the optimal visibility of both the virtual and the real trajectory drawn gradually with the pencil during the execution of the AR-guided task, thus avoiding that the line traced by the user become occluded, to some extent, by holograms (**Figure 7**). Push buttons and voice commands allow to control the appearance and disappearance of the virtual objects, thus facilitating the optimal visibility of relevant elements during the task (e.g. the skin layer used as Model Target for marker-less registration can be removed after having checked the achievement of a good registration).

For tests using tablet, the mobile device was anchored to an articulated arm fixed to the table on which the leg phantom was placed. By means of the articulated arm the tablet can be oriented and locked in the most appropriate position to allow the correct visualization of the holographic overlay, and the user to freely carry out the task.

For tests using HoloLens 2, a preliminary user experience with the mixed reality headset was provided, also including the calibration procedure which is required to ensure the best hologram viewing experience for each subject.

Each subject was instructed that the primary goal of the test was to accurately trace a line following the virtual skin paddle profile displayed in AR (see **Suppl_Video_3_Tablet_TASK**), both through the tablet, and through the HoloLens 2 smart glasses.

For both test sessions, the AR-guided task was performed after the tracking of leg phantom profile has been achieved, and the optimal registration between the holographic virtual content and the real phantom has been visually checked and verified using the cutting guide.

**Figure 8:**
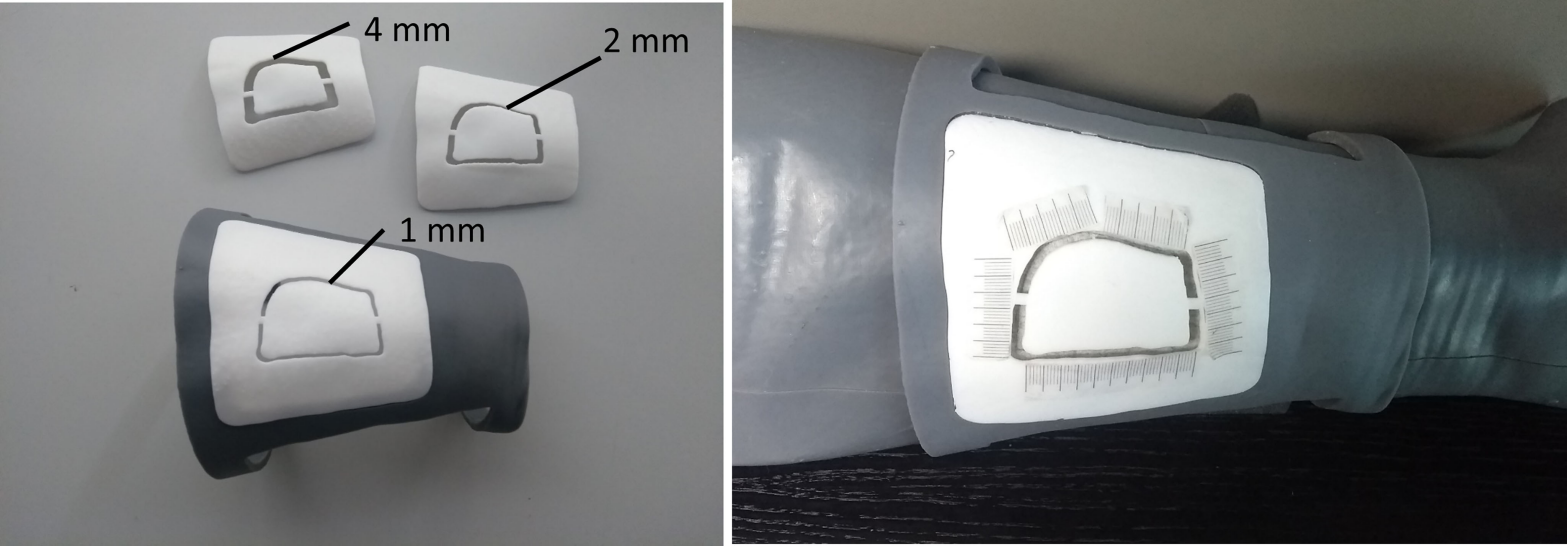
CAD/CAM templates used to evaluate the success rate in performing the AR-guided task, using different levels of accuracy (template “1 mm”, template “2 mm”, template “4 mm”).

A 0.5 mm pencil was used to draw the perceived profile on an adhesive tape applied over the leg phantom surface. The success rate in tracing the AR-displayed profile was evaluated using CAD/CAM templates to be positioned on the surface of the phantom model using a similar setting adopted in our previous works (14, 16). Each template for skin paddle outline can be uniquely positioned on the leg phantom as it is a customized CAD/CAM design starting from the leg model reconstructed from CT scan. The anatomical fitting of each template on phantom calf is obtained thanks to the shape of the template itself: two customized flanges embrace circumferentially the proximal and distal portions of the leg, allowing to position it univocally in all dimensions (**Figure 8**).

The templates were 3D printed (Form 3, Formlabs) with a groove of different widths (4 mm, 2 mm, 1 mm) in order to evaluate three levels of achievable accuracy: ± 2.0 mm, ± 1.0 mm, and ± 0.5 mm (**Figure 8**). A millimeter adhesive tape was associated to each template and used to measure the cumulative length of the traced skin paddle profile falling within the groove, and then to calculate the percentage of successful traced trajectories (“percentage success rate”) (**Figure 8**). We considered as successful competition of the task (100% success rate) those trials in which the traced skin paddle profile fell within the groove of the cutting guide along its entire length (16.5 cm).

### 2.3 Statistics

All results about the registration errors (Test Session 1) and the percentage of success rate in performing the AR-guided task (Test Session 2) were reported as mean values and standard deviation (SD).

For Test Session 1, T-test for unpaired data was used to compare the mean registration errors, both in X and Y direction, for the tablet-based and HoloLens-based groups, as well as to evaluate for each AR display type the difference of mean values between the “OFF_lamp” and “ON_Lamp” lightening condition.

For Test Session 2, the difference of the mean percentage success rate obtained with the two AR display types was evaluated using T-test for paired samples.

SPSS software (IBM, Armonk, New York, US) was used to perform the statistical analysis, and a p value of <0.05 was considered statistically significant.

## 3 Results

**Figure 9:**
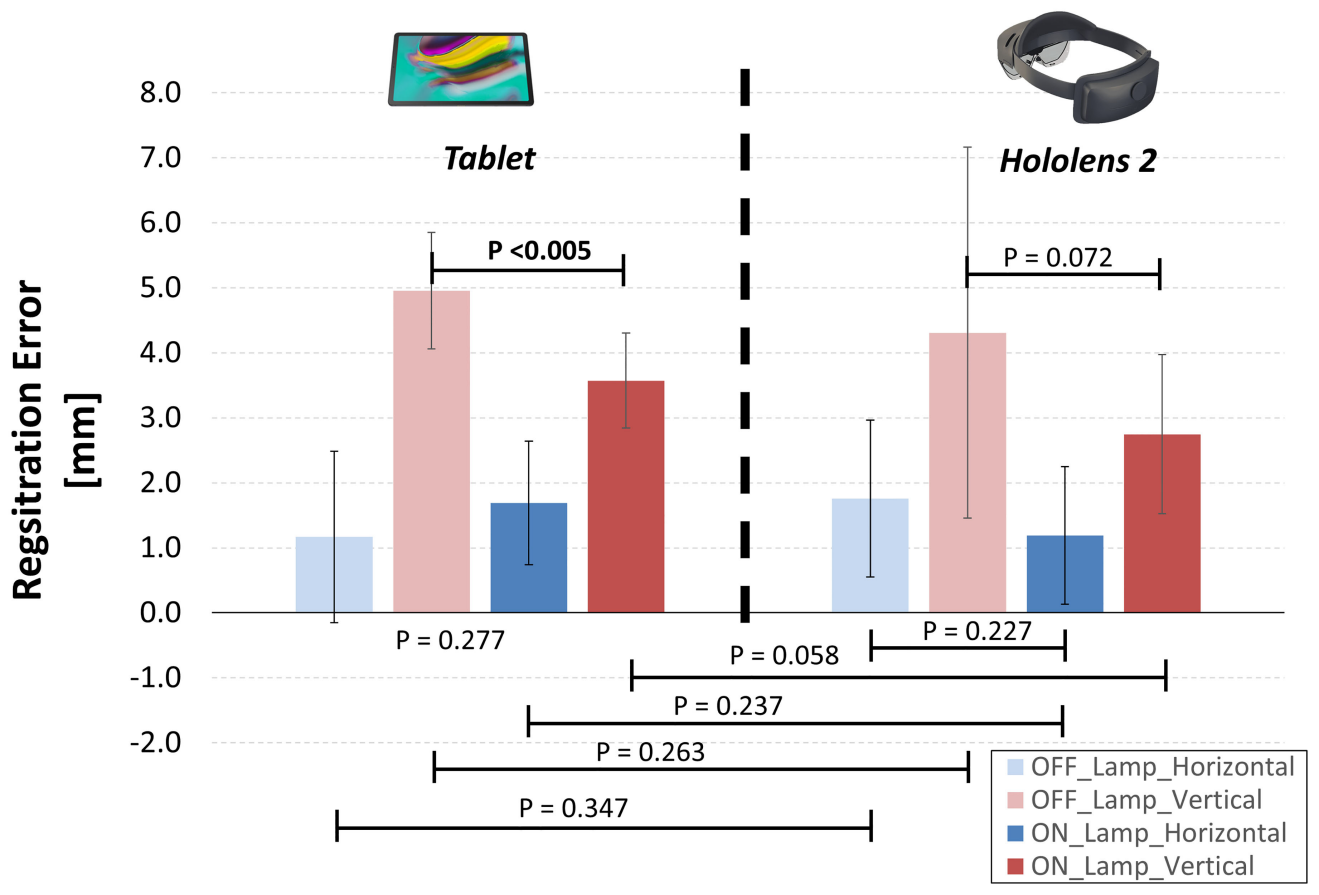
Resulting registration errors calculated as mean horizontal and vertical deviations between the real and virtual content, for two lighting conditions: environment illuminated by natural daylight (OFF_Lamp); artificial lamp light that points directly on the phantom (ON_Lamp).

On average, we found a similar registration errors for tablet-based and HoloLens-based solutions, ranging between 1-5 mm (**Figure 9**). The largest deviations between virtual and real content were in the vertical (Y) direction for “OFF_lamp” lightening condition, while lower registration errors (within 2 mm) resulted in the horizontal (X) direction (blue bars in **Figure 9**).

For tablet-based application, the “ON-Lamp” lightening condition provided a statistically significant reduction of the registration error in the vertical direction if compared with “OFF-lamp” condition (3.6 ± 0.7 mm vs 5.0 ± 0.9 mm, p <0.005), while the registration error in horizontal direction did not significantly change. The “ON-Lamp” lightening condition seems to reduce also the registration error for Hololens-based application, particularly in vertical direction, although this reduction did not result statistically significant (2.7 ± 1.2 mm vs 4.2 ± 2.4 mm, p = 0.072).

Results from Test Session 2 are summarized in **Table 1**. With HoloLens 2 all subjects (100%) were able to successfully trace the skin paddle profile with an accuracy level of ±2.0 mm (verified with the “4 mm” template); with tablet, on average, the 97% of the traced trajectories was within ±2.0 mm accuracy level.

**Table 1:** Resulting mean percentage success rate in performing the AR-guided task using both tablet and HoloLens 2, for different accuracy levels (“1 mm”, “2 mm”, “4 mm” templates).

Subject	Tablet			HoloLens 2		
	Template 1 mm ( ± 0.5 mm)	Template 2 mm ( ± 1 mm)	Template 4 mm ( ± 2 mm)	Template 1 mm ( ± 0.5 mm)	Template 2 mm ( ± 1 mm)	Template 4 mm ( ± 2 mm)
1	30%	48%	100%	33%	94%	100%
2	15%	48%	91%	28%	36%	100%
3	6%	76%	100%	21%	45%	100%
4	24%	45%	100%	45%	91%	100%
5	21%	52%	100%	48%	82%	100%
6	27%	42%	100%	30%	42%	100%
7	3%	30%	82%	76%	97%	100%
8	24%	79%	100%	45%	82%	100%
**Mean**	**19%**	**53%**	**97%**	**41%**	**71%**	**100%**
SD	10%	17%	7%	17%	25%	0%

Bold values are used to identify/highlight the mean values.

For accuracy level ±1 mm and ±0.5 mm, lower success rates resulted for both tablet and HoloLens solutions (53%, 19% and 71%, 41%, respectively), (**Table 1**). We found no statistically significant difference in success rate between tablet-based and HoloLens-based AR application, except for ±0.5 mm accuracy level, where AR guidance with HoloLens 2 showed a success rate higher than the one achievable with tablet (p<0.05).

From Test Session 1 we realized that the implemented marker-less registration is quite sensitive to environment lightening conditions, and that holograms can appear to project accurately over the leg phantom only from a certain perspective (i.e. a quite frontal perspective corresponding to the one chosen for Model Target creation in Vuforia Engine), whereas from other perspectives the hologram revealed very inaccurate placement.

In performing Test Session 2, we observed some inherent drawbacks of the marker-less registration based on the recognition of the 3D object profile using Vuforia Engine Model Target function. Especially in tablet-based application we observed that the hologram tends to shift away a little bit from the optimal registration position as soon as the subject approaches the hand to the phantom to start drawing the AR-guided trajectory. This behavior may be due to the fact that the subject places his hand between the camera and the tracked real object, thus interfering with the correct recognition of the skin layer profile of the leg phantom used as Model Target.

In terms of user experience, all subjects reported greater usability and ergonomics of the HoloLens solution, although the brightness of the virtual content was lower than the one displayed on the tablet.

## 4 Discussion

AR is a promising technology for craniofacial surgeons to obtain a “see-through” effect in the operating room. However, some problems with AR, such as depth perception (27) and registration errors (28), i.e. the difference between virtual content and actual reality resulting in bias for users, still remain.

The present study aims to increase the information regarding the achievable registration accuracy with a marker-less AR application usable *via* a tablet and a HoloLens 2 headset, that we developed for assisting skin paddle harvesting in osteomyocutaneous fibular flap procedure for mandibular reconstruction. The perspective of clinical application of the proposed AR protocol for head and neck reconstruction is the 3D planning of the soft tissue resection and reconstruction, having the opportunity to reduce the donor site morbidity, improving eventually the three-dimensional reconstructive outcome.

From a technical point of view, the primary challenge that needs to be addressed for AR to become a viable tool for surgery is the accuracy of registration between the displayed virtual content and the real scene. The registration error, mainly caused by registration method and camera performance, results in a “misalignment” effect in the subjective perception for virtual and reality image. The registration error is divided into static registration error and dynamic registration error. The static registration error deals with the error caused by the system when the user’s viewpoint is still at rest with the real object, whereas the dynamic registration error refers to the error that occurs when the real object has relative motion in the user’s view or environment (29). In our study we quantified the static registration error obtained for both tablet and HoloLens solutions, when using a marker-less tracking method based on the recognition of the 3D object profile.

Most of the current AR approaches are limited to invasive marker fixation to provide virtual-to-patient registration. In craniomaxillofacial surgery, AR solutions based on a marker-less tracking, like the one we propose, may offer the advantage to overcome the inherent drawbacks of standard navigation systems, such as the use of reference marks to be positioned on the patient, and the need for a quite long registration procedure. Therefore, a marker-less tracking approach offers a less intrusive solution and limits the need for manufacturing dedicated CAD/CAM trackers anchored for example to occlusal splints, which may obstruct the operative field.

On the other hand, marker-less solutions, as we observed in our experiments, may suffer from poor robustness, being sensitive to ambient light conditions, to changes in viewpoint, to contrast image and to the integrity of the 3D object profile used as Model Target. Indeed, in our previous experience in real clinical setting (23) we observed evident registration errors due to the posterior leg soft tissue displacing after the skin incision, since this surgical action alters the correct recognition of the leg profile used for registration. In general, while hard tissues can be registered with a high degree of success, the ability to accurately track mobile or deformable anatomy still remains a challenge, since soft tissue deformations during intraoperative maneuver reduce the stability of AR tracking which is based on a static rigid 3D model.

In the present study we found mean registration errors ranging within 1-5 mm, for both tablet-based and HoloLens-based solution, considering two dimensions, i.e. horizontal (X) and vertical (Y) directions. Our findings were quite in line with other studies on HoloLens accuracy for surgical applications that reported visualization errors up around 2 millimeters (30–32) and more than 5 millimeters in one case (33).

We observed largest deviations in the vertical (Y) direction. This may be due to the “Model Target” registration function which is based on recognition of the shape of a 3D object (i.e. the leg) observed from a certain perspective. In horizontal direction (X) there is an excellent fitting between the real and virtual content since the proximal and distal edge of the leg are correctly recognized and matched; however, the virtual-to-real matching with the Model Target function can occur also with a persisting rotational component along the leg long axis; this leads to a more relevant misalignment of the projected skin paddle profile in the vertical direction (Y).

Typically, the skin paddle used for reconstruction is a soft tissue area of average 6x4cm^2^ at least, so a sub-millimetric accuracy in virtual-to-real registration is not required and even an error of 5-10 millimeters can be acceptable, since it does not affect the clinical outcome. Therefore, our findings are satisfying in terms of accuracy since the resulting average registration errors are absolutely compatible with the objectives and clinical applications of the proposed AR protocol.

For marker-less tracking we chose the external profile of the leg skin as Model Target to be used in Vuforia Engine package; this means that all the virtual structures of interest to be projected in AR (fibula, arterial vessels, skin paddle profile) are included in the chosen Model Target. This could be a favorable aspect to minimize the registration error, since the farther the augmented virtual object is from the object used for tracking, the greater the registration error.

For the AR-guided task we found a good success rate (around 100%) in completing the task within an error margin of 4 mm for both AR display solutions. These results have to take into account that for each test we started from a condition of optimal registration error (around 2 mm), which was visually checked before the subject started tracing the trajectory under the AR guidance, in order to maximize the achievable success rate.

Regarding AR display types, HMDs are emerging as efficient and promising media to support complex manual surgical tasks typically performed under direct vision (8, 34), since they allow the surgeon to maintain a “surgeon-centered’ point of view and to leave his/her hands free to operate on the patient. Nowadays, optical-see-through HMD, like Microsoft HoloLens 2 smart glasses, are the leading wearable AR technology that are being explored also for applications in surgery. Nevertheless, technological and human-factor limitations, such as the small augmentable field of view, the low contrast image, and the still limited registration accuracy for high-precision surgical tasks, still hinder their routine use.

This does not exclude that they can be useful and usable, as in the case of the present study, to assist surgical procedures where there are no stringent accuracy requirements (i.e. submillimetric accuracy). Indeed, the expectation for AR system accuracy should be commensurated with the surgical tasks for which the tool is intended. In our study, all the performed AR-guided tracings on phantom encompassed appropriate skin regions to include the planned perforator vessel for fibular flap harvesting, also in those cases where low percentage of success rate resulted from accuracy verification through CAD/CAM templates.

When surgeons need to accomplish extremely delicate procedures such as precise drilling or cutting in narrow operative areas, AR systems specifically designed for high-precision surgical tasks, i.e. capable of guaranteeing a submillimetric accuracy level, should be preferred (8, 14, 35, 36).

From our experience, comparing the tablet-based and the HoloLens-based solution the following pros and cons emerged. The major advantage of a tablet solution is the good brightness of the virtual rendered anatomy. On the other hand, the advantage of a “surgeon-centered’ point of view is lost, and it is necessary to fix the mobile device in a suitable position that allows the AR view for the surgeon and at the same time the possibility to perform manually the AR-guided surgical task.

Regarding HoloLens 2 we received feedbacks from users of a quite comfortable and ergonomics headset, that offers the advantages of optical see-through technology and hands-free operation. However, the provided contrast image is quite low.

The preoperative planning time for the proposed AR protocol is about 3 hours: 2 hours for CT image segmentation and virtual content preparation, if good quality imagining is provided, and 1 hour for AR application development and its deployment on HoloLens smart glasses. This time would not represent a limitation for clinical use. Indeed, the proposed AR protocol for skin paddle incision can be used as an alternative to CAD/CAM approach based on skin paddle outline guides that require a comparable or even longer preoperative planning time (if we consider also the 3D printing time). Moreover, the AR technology, implemented in a mark-less way, has the great potential of being a “streamlined”, non-obstructive technology, which can be easily transferred and applied in the surgical setting.

Our study has some limitations. In Test Session 1 we quantified only the static registration error, while dynamic misalignment that occurs when the leg phantom has relative motion in the user’s view or environment was not evaluated.

Moreover, in Test Session 2, each subject performed the task consecutively using the two different AR display types, so in the second execution of the same task he/she could have benefited from a little training effect. We tried to limit this bias, inverting the order of execution of the test with tablet and HoloLens between one subject and another.

As future development, we plan to enriched the AR based protocol with additional features that allow the simultaneous tracking of multiple 3D objects, e.g. a target anatomical region and a movable bone segment to be repositioned or displayed relative to the target. This may open the way to exploring new surgical or outpatient applications of augmented reality in the craniomaxillofacial field.

Future perspective will address to transfer the proposed AR protocol in the real clinical field to assist the skin paddle harvesting in osteomyocutaneous fibular flap reconstructive surgery. Surely, the head mounted display such as the HoloLens 2 solution is the most promising and viable option for use in the operating room setting. So, our future efforts will be focused on promoting a clinical study on the use of the AR protocol with HoloLens 2 headset.

## 5 Conclusions

In this study we present the development of a marker-less AR based protocol proposed to assist skin paddle harvesting in osteomyocutaneous fibular flap procedure for mandibular reconstruction. The developed AR guidance system was evaluated on a 3D printed leg phantom, thus allowing a systematic comparison of the achievable static registration accuracy and of the success rate in performing the AR-guided skin paddle harvesting task, when using two different AR display solutions, i.e. a tablet and HoloLens 2 headset. Results revealed that the AR based protocol provides a registration error within the range 1-5 mm for assisting skin paddle harvesting in the clinical setting, in both solutions. Greater usability and ergonomics resulted for HoloLens 2. Optimal lightening conditions and further improvement of marker-less tracking technologies have the potential to increase the efficiency and precision of this AR-assisted reconstructive surgery.

## Data Availability Statement

The raw data supporting the conclusions of this article will be made available by the authors, without undue reservation.

## Ethics Statement

The studies involving human participants were reviewed and approved by the Ethical Committee of IRCCS Hospital - University of Bologna, Policlinico S. Orsola-Malpighi (protocol no: 62/Sper/AOUBo, Comitato Etico Area Vasta Emilia Centro CE-AVEC). The patients/participants provided their written informed consent to participate in this study.

## Author Contributions

LC: study concept and design, manuscript preparation, and dataanalysis. FB: software development, and data collection and analysis. GB, SB, and AT: study design and data interpretation. CM: study supervision. EM: study concept and study supervision. LC, GB, AT, CM, SB, and EM: contributed to the manuscript review. All authors contributed to the article and approved the submitted version.

## Conflict of Interest

The authors declare that the research was conducted in the absence of any commercial or financial relationships that could be construed as a potential conflict of interest.

## Publisher’s Note

All claims expressed in this article are solely those of the authors and do not necessarily represent those of their affiliated organizations, or those of the publisher, the editors and the reviewers. Any product that may be evaluated in this article, or claim that may be made by its manufacturer, is not guaranteed or endorsed by the publisher.

## Supplementary Material

Video 1 Use of the AR application on the phantom, via tablet.

Video 2 Use of the AR application on the phantom, via HoloLens 2 smart glasses.

Video 3 Execution of the AR-guided task on phantom.

## References

[1] Chidambaram S Stifano V Demetres M Teyssandier M Palumbo MC Redaelli A. Applications of Augmented Reality in the Neurosurgical Operating Room: A Systematic Review of the Literature. J Clin Neurosci (2021) 91:43–61. doi: 10.1016/j.jocn.2021.06.032 34373059

[2] Hersh A Mahapatra S Weber-Levine C Awosika T Theodore JN Zakaria HM. Augmented Reality in Spine Surgery: A Narrative Review. HSS J (2021) 17(3):351–8. doi: 10.1177/15563316211028595 PMC8436352 34539277

[3] Reis G Yilmaz M Rambach J Pagani A Suarez-Ibarrola R Miernik A. Mixed Reality Applications in Urology: Requirements and Future Potential. Ann Med Surg (2021) 66:102394. doi: 10.1016/j.amsu.2021.102394 PMC8141462 34040777

[4] Schiavina R Bianchi L Lodi S Cercenelli L Chessa F Bortolani B. Real-Time Augmented Reality Three-Dimensional Guided Robotic Radical Prostatectomy: Preliminary Experience and Evaluation of the Impact on Surgical Planning. Eur Urol Focus (2021) 7(6):1260–7. doi: 10.1016/j.euf.2020.08.004 32883625

[5] Schiavina R Bianchi L Chessa F Barbaresi U Cercenelli L Lodi S. Augmented Reality to Guide Selective Clamping and Tumor Dissection During Robot-Assisted Partial Nephrectomy: A Preliminary Experience. Clin Genitourin Cancer (2021) 19(3):e149–55. doi: 10.1016/j.clgc.2020.09.005 33060033

[6] Verhey JT Haglin JM Verhey EM Hartigan DE. Virtual, Augmented, and Mixed Reality Applications in Orthopedic Surgery. Int J Med Robot (2020) 16(2):1–9. doi: 10.1002/rcs.2067 31867864

[7] Matthews JH Shields JS. The Clinical Application of Augmented Reality in Orthopaedics: Where Do We Stand? Curr Rev Musculoskelet Med (2021) 14(5):316–9. doi: 10.1007/s12178-021-09713-8 PMC8497656 34581989

[8] Badiali G Cercenelli L Battaglia S Marcelli E Marchetti C Ferrari V. Review on Augmented Reality in Oral and Cranio-Maxillofacial Surgery: Toward “Surgery-Specific” Head-Up Displays. IEEE Access (2020) 8:59015–28. doi: 10.1109/ACCESS.2020.2973298

[9] Benmahdjoub M van Walsum T van Twisk P Wolvius EB. Augmented Reality in Craniomaxillofacial Surgery: Added Value and Proposed Recommendations Through a Systematic Review of the Literature. Int J Oral Maxillofac Surg (2021) 50(7):969–78. doi: 10.1016/j.ijom.2020.11.015 33339731

[10] Mezger U Jendrewski C Bartels M. Navigation in Surgery. Langenbecks Arch Surg (2013) 398(4):501–14. doi: 10.1007/s00423-013-1059-4 PMC3627858 23430289

[11] Molina CA Sciubba DM Greenberg JK Khan M Witham T. Clinical Accuracy, Technical Precision, and Workflow of the First in Human Use of an Augmented-Reality Head-Mounted Display Stereotactic Navigation System for Spine Surgery. Oper Neurosurg Hagerstown Md (2021) 20(3):300–9. doi: 10.1093/ons/opaa398 33377137

[12] Yahanda AT Moore E Ray WZ Pennicooke B Jennings JW Molina CA. First in-Human Report of the Clinical Accuracy of Thoracolumbar Percutaneous Pedicle Screw Placement Using Augmented Reality Guidance. Neurosurg Focus (2021) 51(2):E10. doi: 10.3171/2021.5.FOCUS21217 34333484

[13] Kiarostami P Dennler C Roner S Sutter R Fürnstahl P Farshad M. Augmented Reality-Guided Periacetabular Osteotomy-Proof of Concept. J Orthop Surg (2020) 15(1):540. doi: 10.1186/s13018-020-02066-x PMC7672946 33203429

[14] Cercenelli L Carbone M Condino S Cutolo F Marcelli E Tarsitano A. The Wearable VOSTARS System for Augmented Reality-Guided Surgery: Preclinical Phantom Evaluation for High-Precision Maxillofacial Tasks. J Clin Med (2020) 9(11):E3562. doi: 10.3390/jcm9113562 33167432 PMC7694536

[15] Glas HH Kraeima J van Ooijen PMA Spijkervet FKL Yu L Witjes MJH. Augmented Reality Visualization for Image-Guided Surgery: A Validation Study Using a Three-Dimensional Printed Phantom. J Oral Maxillofac Surg (2021) 79(9):1943.e1–1943.e10. doi: 10.1016/j.joms.2021.04.001 34033801

[16] Condino S Fida B Carbone M Cercenelli L Badiali G Ferrari V. Wearable Augmented Reality Platform for Aiding Complex 3d Trajectory Tracing. Sensors (2020) 20(6):E1612. doi: 10.3390/s20061612 32183212 PMC7146390

[17] Kumar BP Venkatesh V Kumar KAJ Yadav BY Mohan SR. Mandibular Reconstruction: Overview. J Maxillofac Oral Surg (2016) 15(4):425–41. doi: 10.1007/s12663-015-0766-5 PMC5083680 27833334

[18] Futran ND Stack BC Zaccardi MJ. Preoperative Color Flow Doppler Imaging for Fibula Free Tissue Transfers. Ann Vasc Surg (1998) 12(5):445–50. doi: 10.1007/s100169900182 9732422

[19] Goetze E Kämmerer PW Al-Nawas B Moergel M. Integration of Perforator Vessels in CAD/CAM Free Fibula Graft Planning: A Clinical Feasibility Study. J Maxillofac Oral Surg (2020) 19(1):61–6. doi: 10.1007/s12663-019-01215-y PMC6954946 31988566

[20] González Martínez J Torres Pérez A Gijón Vega M Nuñez-Villaveiran T. Preoperative Vascular Planning of Free Flaps: Comparative Study of Computed Tomographic Angiography, Color Doppler Ultrasonography, and Hand-Held Doppler. Plast Reconstr Surg (2020) 146(2):227–37. doi: 10.1097/PRS.0000000000006966 32740566

[21] Battaglia S Maiolo V Savastio G Zompatori M Contedini F Antoniazzi E. Osteomyocutaneous Fibular Flap Harvesting: Computer-Assisted Planning of Perforator Vessels Using Computed Tomographic Angiography Scan and Cutting Guide. J Cranio-Maxillo-fac Surg (2017) 45(10):1681–6. doi: 10.1016/j.jcms.2017.07.017 28838837

[22] Battaglia S Ricotta F Maiolo V Savastio G Contedini F Cipriani R. Computer-Assisted Surgery for Reconstruction of Complex Mandibular Defects Using Osteomyocutaneous Microvascular Fibular Free Flaps: Use of a Skin Paddle-Outlining Guide for Soft-Tissue Reconstruction. A Technical Report. J Cranio-Maxillo-fac Surg (2019) 47(2):293–9. doi: 10.1016/j.jcms.2018.11.018 30558999

[23] Battaglia S Badiali G Cercenelli L Bortolani B Marcelli E Cipriani R. Combination of CAD/CAM and Augmented Reality in Free Fibula Bone Harvest. Plast Reconstr Surg Glob Open (2019) 7(11):e2510. doi: 10.1097/GOX.0000000000002510 31942302 PMC6908345

[24] Battaglia S Ratti S Manzoli L Marchetti C Cercenelli L Marcelli E. Augmented Reality-Assisted Periosteum Pedicled Flap Harvesting for Head and Neck Reconstruction: An Anatomical and Clinical Viability Study of a Galeo-Pericranial Flap. J Clin Med (2020) 9(7):E2211. doi: 10.3390/jcm9072211 32668591 PMC7408700

[25] Linte CA Davenport KP Cleary K Peters C Vosburgh KG Navab N. On Mixed Reality Environments for Minimally Invasive Therapy Guidance: Systems Architecture, Successes and Challenges in Their Implementation From Laboratory to Clinic. Comput Med Imaging Graph (2013) 37(2):83–97. doi: 10.1016/j.compmedimag.2012.12.002 23632059 PMC3796657

[26] Pérez-Pachón L Poyade M Lowe T Gröning F. Image Overlay Surgery Based on Augmented Reality: A Systematic Review. Adv Exp Med Biol (2020) 1260:175–95. doi: 10.1007/978-3-030-47483-6_10 33211313

[27] Bremers AWD Yöntem AÖ Li K Chu D Meijering V Janssen CP. Perception of Perspective in Augmented Reality Head-Up Displays. Int J Hum-Comput Stud (2021) 155:102693. doi: 10.1016/j.ijhcs.2021.102693

[28] Tokunaga DM Corrêa CG Bernardo FM Bernardes J Ranzini E Nunes FLS. Registration System Errors Perception in Augmented Reality Based on RGB-D Cameras. In: Shumaker R Lackey S, editors. Virtual, Augmented and Mixed Reality. Cham: Springer International Publishing (2015). p. 119–29. Lecture Notes in Computer Science.

[29] Jiang J Huang Z Qian W Zhang Y Liu Y. Registration Technology of Augmented Reality in Oral Medicine: A Review. IEEE Access (2019) 7:53566–84. doi: 10.1109/ACCESS.2019.2912949

[30] Moreta-Martinez R García-Mato D García-Sevilla M Pérez-Mañanes R Calvo-Haro J Pascau J. Augmented Reality in Computer-Assisted Interventions Based on Patient-Specific 3D Printed Reference. Healthc Technol Lett (2018) 5(5):162–6. doi: 10.1049/htl.2018.5072 PMC6222179 30464847

[31] Liu H Auvinet E Giles J Rodriguez Y Baena F. Augmented Reality Based Navigation for Computer Assisted Hip Resurfacing: A Proof of Concept Study. Ann BioMed Eng (2018) 46(10):1595–605. doi: 10.1007/s10439-018-2055-1 PMC6153979 29796955

[32] Meulstee JW Nijsink J Schreurs R Verhamme LM Xi T Delye HHK. Toward Holographic-Guided Surgery. Surg Innov (2019) 26(1):86–94. doi: 10.1177/1553350618799552 30261829

[33] Condino S Carbone M Piazza R Ferrari M Ferrari V. Perceptual Limits of Optical See-Through Visors for Augmented Reality Guidance of Manual Tasks. IEEE Trans BioMed Eng (2020) 67(2):411–9. doi: 10.1109/TBME.2019.2914517 31059421

[34] Ferrari V Carbone M Condino S Cutolo F. Are Augmented Reality Headsets in Surgery a Dead End? Expert Rev Med Devices (2019) 16(12):999–1001. doi: 10.1080/17434440.2019.1693891 31725347

[35] Badiali G Cutolo F Cercenelli L Carbone M D’Amato R Ferrari V. The Vostars Project: A New Wearable Hybrid Video and Optical See-Through Augmented Reality Surgical System for Maxillofacial Surgery. Int J Oral Maxillofac Surg (2019) 48:153. doi: 10.1016/j.ijom.2019.03.472

[36] Cutolo F Freschi C Mascioli S Parchi PD Ferrari M Ferrari V. Robust and Accurate Algorithm for Wearable Stereoscopic Augmented Reality With Three Indistinguishable Markers. Electronics (2016) 5(3):59. doi: 10.3390/electronics5030059

